# Pathophysiological insights and therapeutic developments in age-related hearing loss: a narrative review

**DOI:** 10.3389/fnagi.2025.1657603

**Published:** 2025-08-20

**Authors:** Shichu Sun, Qi Zhao, Haojia He, Yujia Liu, Yuchong Nie, You Zhou

**Affiliations:** ^1^Laboratory of Sensory Neurobiology, School of Basic Medical Sciences, Hebei University, Baoding, China; ^2^Key Laboratory of Aging and Health in Hebei, Baoding, China

**Keywords:** age-related hearing loss, presbycusis, pathogenesis, drug targets, therapeutics

## Abstract

Age-related hearing loss (ARHL), or presbycusis, is characterized by a progressive decline in binaural auditory sensitivity, particularly affecting high-frequency hearing and sound localization. The pathogenesis of ARHL is still unclear, correspondingly reflected in a lack of clinically effective intervention strategies. Recent advancements in audiology and neurobiology have illuminated the black box of the pathogenesis of ARHL. The intricate mechanisms underlying ARHL involve inflammation, oxidative stress, excessive autophagy, cellular signaling dysregulation, and metabolic alterations, which cause substantial damage to cellular function within cochlea. The weakened sound conduction and reduced auditory information processing potentially lead to emotional distress and heightened susceptibility to neurodegenerative conditions like cognitive decline and dementia. Promising interventions targeting these mechanisms are actively being investigated, ranging from pharmacological approaches to genetic therapies and lifestyle interventions. This narrative review summarizes recent research progress in understanding ARHL pathogenesis and discusses emerging strategies for prevention and treatment, highlighting the imperative for targeted interventions to enhance auditory health and overall well-being in aging populations.

## Graphical Abstract

Schematic representation of the proposed mechanisms underlying age-related hearing loss (ARHL), involving inflammation, oxidative stress, excessive autophagy, dysregulated cellular signaling, and metabolic alterations within the cochlea, leading to progressive auditory decline and increased vulnerability to neurodegenerative conditions, alongside emerging pharmacological, genetic, and lifestyle-based intervention strategies. Created with Biorender.com. 
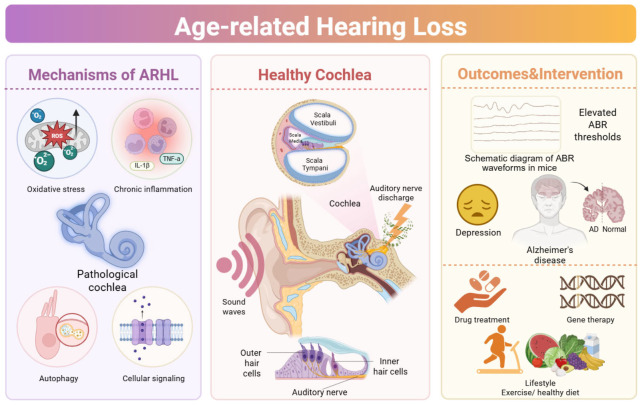

## 1 Introduction

Aging is associated with a decline in the function of multiple organ systems, inner ear is particularly susceptible to the effects of aging (López-Otín et al., 2023). With advancing age, the structure and function of inner ear deteriorate irreversibly, resulting in progressive bilateral sensorineural hearing loss, primarily affecting high-frequency hearing. This condition, commonly referred to as age-related hearing loss (ARHL) or presbycusis. Individuals with ARHL often face challenges in speech discrimination and experience difficulties in sound detection and localization, especially in noisy environments (Bowl and Dawson, 2019). ARHL is the most prevalent sensory deficit among older adults and often leads to social isolation, as well as comorbidities such as frailty, falls, late-onset depression, cognitive decline, and dementia (Lin et al., 2013; Loughrey et al., 2018; Rutherford et al., 2018). According to the World Health Organization, an estimated 2.5 billion people will be affected by hearing loss, with over 500 million individuals experiencing significant impairment due to ARHL (Chadha et al., 2021; GBD 2019 Hearing Loss Collaborators, 2021). Despite its high prevalence, effective preventive and therapeutic strategies for ARHL remain limited, and no specific otoprotective agents have been approved for this condition. Currently, the commonly used and effective intervention is the passive use of hearing aids, however, this approach does not fully address the needs of all ARHL patients (Li-Korotky, 2012). Thus, a comprehensive exploration of the mechanisms underlying the development of ARHL and the identification of targeted treatment strategies are crucial for improving the quality of life for the elderly.

**FIGURE 1:**
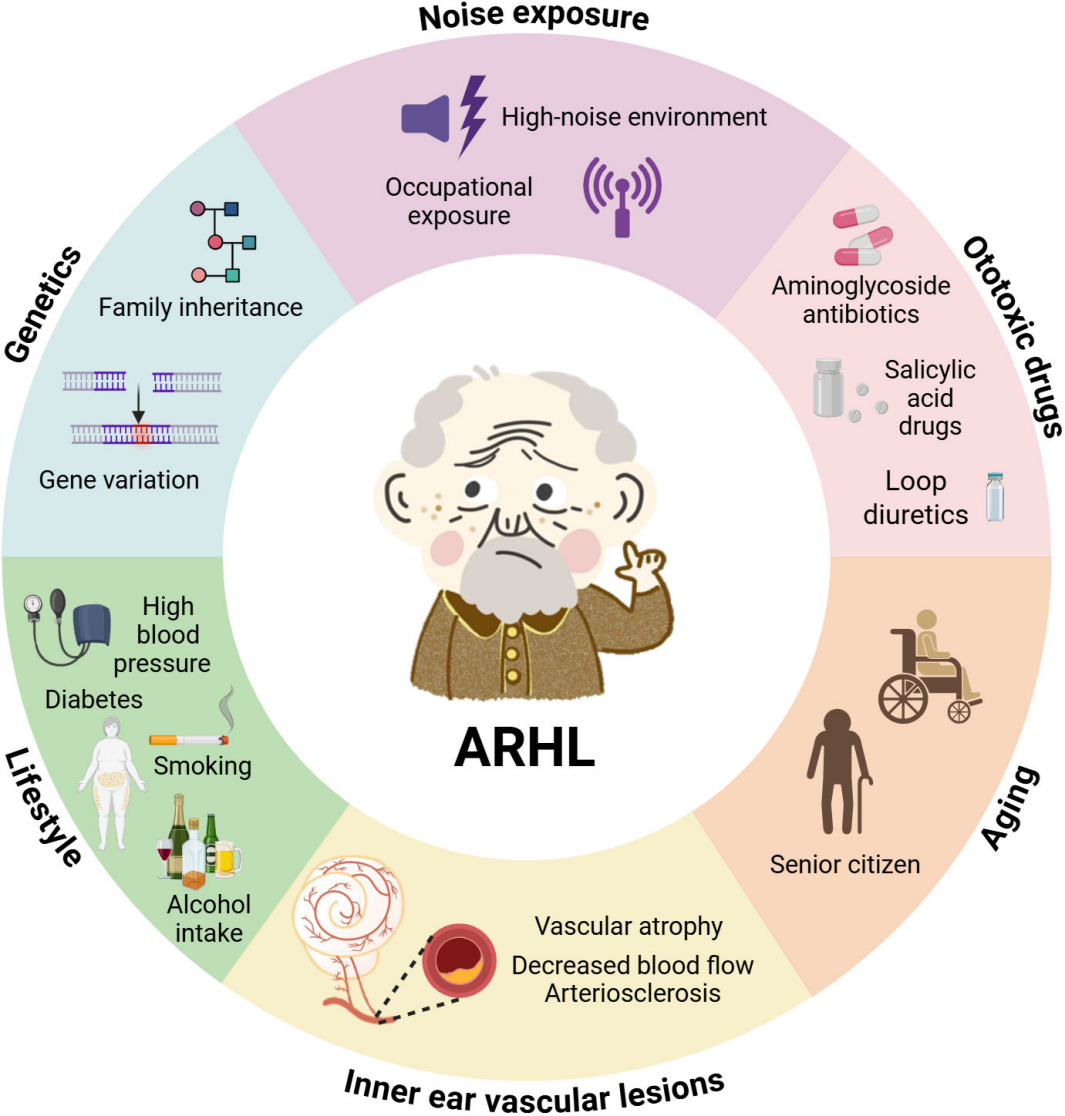
The interplay of endogenous and exogenous factors accelerates auditory senescence. Age-related hearing loss (ARHL) arises from a multifactorial interplay between intrinsic (endogenous) and extrinsic (exogenous) influences. Endogenous factors include genetic predisposition (e.g., gene variants, family inheritance) and age-related degeneration. Exogenous contributors encompass environmental noise exposure (e.g., occupational or high-noise environments), ototoxic medications (such as aminoglycoside antibiotics, salicylates, and loop diuretics), vascular pathology within the inner ear (e.g., decreased blood flow, vascular atrophy, arteriosclerosis), and lifestyle-related conditions (including diabetes, hypertension, smoking, and alcohol intake). Together, these factors contribute synergistically to the onset and progression of ARHL. Created with Biorender.com.

Age-related hearing loss is primarily influenced by genetic factors but is also significantly affected by various environmental factors such as noise exposure, ototoxic medications, heavy metals, and lifestyle choices including smoking, alcohol consumption, and diet, as well as metabolic diseases (Bowl and Dawson, 2019; Figure 1). These pathological factors lead to irreversible degeneration of various auditory structures, including sensory hair cells, primary afferent neurons, stria vascularis, lateral wall, and the central auditory system, all of which are believed to be involved in ARHL (Yang et al., 2015; Tawfik et al., 2020; Eckert et al., 2021; Lang et al., 2023). Although the underlying mechanisms are still under investigation, accumulating evidence supports that chronic inflammation, oxidative stress, excessive autophagy, hormonal disorders, and dysfunction of multiple transporters, receptors, and ion channels in the inner ear play significant roles in this process. This narrative review aims to provide a comprehensive perspective by systematically analyzing the latest research advances in ARHL. Our goal is to integrate existing knowledge and explore the etiology, pathophysiology, and emerging treatment strategies of ARHL, while also highlighting the challenges and future research directions in these areas.

## 2 Literature search and synthesis methodology

To gather relevant literature, a non-systematic search strategy was employed across major scientific databases including PubMed, Google Scholar and Web of Science, covering publications up to July 2025. The search strategy involved using the following keyword combinations: (“age-related hearing loss” OR “presbycusis”) AND (“inflammation” OR “oxidative stress” OR “autophagy” OR “mitochondrial DNA mutation” OR “ion transport” OR “neural degeneration” OR “gene expression” OR “epigenetic regulation” OR “hormones” OR “osteoporosis”) for studies related to pathogenesis; (“age-related hearing loss” OR “presbycusis”) AND (“pharmacological” OR “gene therapy” OR “stem cell transplantation” OR “hearing aid” OR “cochlear implant” OR “lifestyle”) for studies on interventions and (“age-related hearing loss” OR “presbycusis”) AND (“Alzheimer’s disease” OR “cognitive” OR “depression” tinnitus) for research related to comorbidities. Inclusion criteria focused on: Peer-reviewed articles written in English, Original research articles and studies providing insights into ARHL pathogenesis or therapeutic approaches. Exclusion criteria included: studies focusing solely on non-age-related hearing loss, case reports, editorials, letters to the editor, and conference abstracts without full data. The included studies were evaluated qualitatively and synthesized narratively to identify consistent findings, emerging themes, and areas of ongoing debate or uncertainty. As a narrative review, no formal quality appraisal or meta-analysis was performed.

## 3 Multifaceted pathogenesis of ARHL

Pathological studies on postmortem human temporal bones by Schuknecht and Gacek (1993) have shown that ARHL involves the degeneration of several auditory structures (Schuknecht, 1964). Based on the patterns of hearing loss and corresponding structural changes, six types of ARHL have been proposed: (1) sensory presbycusis, characterized by a sudden increase in pure-tone threshold at high frequencies and loss of basal hair cells; (2) neural presbycusis, defined by a widespread loss of spiral ganglion neurons throughout the cochlea; (3) metabolic presbycusis, marked by a flat or slightly sloping pure-tone audiogram and atrophy of the stria vascularis; (4) mechanical presbycusis, involves changes in the physical structure and properties of the basilar membrane. Some researchers suggest this subtype represents an extreme case of metabolic presbycusis, as there is little evidence for age-related stiffening of the organ of Corti’ mechanical structure; (5) mixed presbycusis, characterized by multiple pathological change within the cochlea, incorporating features of at least two of the aforementioned types; (6) intermediate presbycusis, shows no apparent pathological changes under light microscopy, though it includes changes in cellular organelles, reduced hair cell synapses, and chemical changes in the endolymph (Schuknecht, 1964; Schuknecht and Gacek, 1993; He et al., 2019). Despite significant progress in understanding ARHL, the exact mechanisms underlying age-related degeneration in cochlear structures remain elusive. This uncertainty stems from the complexity of the individual factors involved and, more critically, from the interplay between various mechanistic pathways that contribute to ARHL. Aging is a gradual and irreversible biological process, typically associated with time-dependent functional declines across organ system. This process is believed to be driven by twelve hallmark processes (López-Otín et al., 2023), which providing possible explanations for the pathophysiological mechanisms behind auditory system aging. Age itself is recognized as a primary causal factor in ARHL, with mounting evidence highlighting its central role in auditory degeneration. Among environmental contributors, chronic noise exposure has emerged as one of the most common and modifiable risk factors. It is known to accelerate cochlear degeneration, often compounding the damage associated with natural aging (Kujawa and Liberman, 2006; Fetoni et al., 2022; Fuentes-Santamaría et al., 2022). Recent evidence supports its role as a major environmental determinant of ARHL (Jung et al., 2024). Consequently, prevention or reduction of noise exposure represents a practical and effective strategy for mitigating ARHL risk. In addition to age and noise exposure, a range of other factors have been implicated in ARHL pathogenesis, including genetic predisposition, chronic inflammation, oxidative stress, excessive autophagy, hormonal imbalances, and dysregulation of cellular signaling pathways. These factors do not act in isolation but instead interact in complex and dynamic ways, underscoring the need for a holistic and integrative approach to understanding and potentially alleviating ARHL.

### 3.1 Genetic predisposition

Heritability studies in humans have estimated that 25%–75% of the variance in ARHL-associated pathology has a genetic component (Karlsson et al., 1997; Gates et al., 1999; Christensen et al., 2001; Viljanen et al., 2007), suggests that ARHL arises from a combination of genetic and environmental factors. Genetic predispositions can influence susceptibility to auditory decline, while environmental factors-such as noise exposure, ototoxic medications, and general health-can further exacerbate the condition. Together, these influences drive the aging process within the auditory system, ultimately leading to reduced hearing acuity and speech perception. The combined influence of genetics and environmental factors obscures their individual contributions to ARHL, but this challenge keeps scientific interest. Friedman et al. (2009) found that the strongest associated gene in ARHL candidate genes is the glutamate metabotropic receptor 7 (GRM7) gene, and several single nucleotide polymorphisms (SNPs) in this gene may be related to the susceptibility of ARHL. Beyond candidate gene studies, genome-wide association studies (GWAS) have revealed a broader genetic architecture. Analyses using datasets from the UK Biobank and two independent Icelandic cohorts identified over 60 novel genetic variants associated with ARHL (Wells et al., 2019; Ivarsdottir et al., 2021).

More recently, a large-scale GWAS meta-analysis involving over 723,000 individuals identified 48 genome-wide significant loci, 10 of which were novel discoveries. A large proportion of these associated loci are missense variants, with half located within regions known to be linked to familial hearing loss. Interestingly, integrating these GWAS findings with single-cell RNA sequencing data from mouse cochleae and brain revealed that genetic association signals are particularly enriched in the cochlear stria vascularis, further supporting the critical role of this structure in the pathogenesis of ARHL (Trpchevska et al., 2022). Moreover, emerging evidence suggests that the genetic basis of ARHL may be influenced by sex differences, pointing to the need for sex-specific analyses in future studies (Lewis et al., 2022).

The expression of ARHL-associated genes varies across cochlear cell subtypes, with the most significantly associated genes showing preferential expression in cochlear hair cells. These genes are enriched in pathways related to apical cell polarity and vesicle trafficking, suggesting that dysfunction and loss of hair cells due to cell death or aging may be one of the mechanisms underlying the development of ARHL (Xue et al., 2021). Notably, ARHL is often caused by the combined action of multiple genetic factors and differs from congenital and early-onset hearing loss, which is typically induced by single-gene mutations. Certain genetic mutations causing hereditary deafness may also be genetic susceptibility factors for ARHL. For example, GJB2 (gap junction protein beta 2) is one of the most common hereditary deafness-causing genes, its p.V37I mutation is closely associated with late-onset progressive hearing loss, especially in East Asian populations (Li et al., 2012). Homozygous or compound heterozygous mutations of p.V37I are associated with progressive hearing loss as individuals age, suggesting that this mutation may be a pathogenic factor for ARHL (Chen et al., 2022). The GJB2 p.V37I point mutation mouse model recapitulates hearing phenotypes similar to those in humans. Dysfunction of gap junction proteins mediates potassium accumulation around hair cells, leading to excitotoxicity in inner hair cells (IHCs), which promotes cochlear pathology and hearing loss (Lin et al., 2019). These findings underscore the importance of comprehensive genetic screening in individuals at risk of ARHL. Early identification of genetic susceptibility can guide preventive strategies and enable targeted interventions, potentially delaying the onset or progression of ARHL.

### 3.2 Chronic inflammation

Chronic inflammation is characterized by a persistent, low-grade inflammatory state marked by the sustained release of pro-inflammatory mediators and progressive tissue damage. Beyond its well-known systemic effects on overall health, chronic inflammation has emerged as a significant contributor to cochlear dysfunction and hearing loss, particularly in the context of ARHL. A growing body of research highlights the multifaceted mechanisms through which chronic inflammation influences auditory decline, underscoring its critical role in the pathogenesis and progression of ARHL. Pro-inflammatory cytokines are central to the initiation and progression of ARHL. These molecules can accelerate apoptosis of cochlear hair cells and supporting cells by activating cell death pathways. For instance, IL-6 has been shown to upregulate Cav1.3 calcium channels via the JAK-MAPK signaling pathway, leading to synaptic damage in inner hair cells and neurotransmitter-induced excitotoxicity, thereby contributing to ARHL (Lu et al., 2024). Additionally, activation of the NF-κB signaling pathway has been implicated in promoting apoptosis and disrupting cochlear homeostasis (Tong et al., 2023). Recent bioinformatics analyses have identified multiple inflammation-related genes that are significantly differentially expressed in ARHL. These genes are involved in key signaling pathways such as the NLRP3 inflammasome and TNF signaling, both of which are closely associated with inflammatory responses and cellular injury, providing further evidence of the tight link between chronic inflammation and ARHL development (Gu et al., 2025). In addition to promoting cellular apoptosis, inflammatory factors also disrupt the cochlear microenvironment, thereby accelerating ARHL. For example, TNF-α impairs cochlear microcirculation, leading to reduced blood supply and compromised auditory function (Ihler et al., 2013). In mouse models of ARHL, CD4^+^ T cells expressing interleukin-1 receptor 2 (IL-1R2) have been found to produce nitric oxide, which contributes to the degeneration of spiral ganglion neurons (SGNs) and compromises the integrity of the blood-labyrinth barrier. Targeted depletion of these immune cell subsets significantly alleviates cochlear inflammation and slows ARHL progression (Parekh and Kaur, 2023).

External factors can further exacerbate chronic inflammation and thus promote ARHL. For instance, a high-fat diet has been shown to induce inflammatory responses in the cochlea (Kociszewska et al., 2021). Age-related changes in the gut microbiota, such as reduced microbial diversity, increased abundance of Gram-negative pro-inflammatory bacteria that produce lipopolysaccharides, and heightened intestinal permeability, facilitate the translocation of microbial components into the bloodstream. These can reach the cochlea via systemic circulation, triggering local inflammation and contributing to ARHL onset (Li et al., 2021; Kociszewska and Vlajkovic, 2022). Chronic inflammation plays a central role in the etiology of ARHL through both intrinsic and extrinsic mechanisms. Lifestyle modifications–such as controlling dietary fat intake and maintaining gut health–may offer promising avenues for mitigating chronic inflammation and thereby slowing the progression of ARHL.

### 3.3 Oxidative stress

Oxidative stress is recognized as a major contributing factor in the pathogenesis of ARHL. The cochlea is a highly energy-demanding organ that depends on mitochondria for energy production through oxidative phosphorylation. During this process, oxygen is reduced to superoxide via the mitochondrial respiratory chain, leading to the generation of reactive oxygen species (ROS). Among the various sources of ROS in the cochlea, NADPH oxidase (NOX) plays a significant role. The NOX family in mammals comprises seven isoforms (NOX1-5, DUOX1, DUOX2), each characterized by distinct expression profiles and physiological functions. Recent studies have reported relatively high mRNA levels of NOX2, NOX3 and NOX4 in both human and mouse cochleae. Elevated NOX activity can modulate genes involved in excitatory signaling pathways, thereby contributing to cochlear damage and the progression of ARHL (Rousset et al., 2020). Among these isoforms, NOX3 is highly and specifically expressed in the inner ear, including IHCs, outer hair cells (OHCs), supporting cells, and spiral ganglion neurons (SGNs) (Bánfi et al., 2004). Its expression is upregulated in response to aging, noise exposure, and ototoxic drugs, positioning NOX3 as a major local source of ROS and a promising target for therapeutic intervention in ARHL (Mohri et al., 2021). In a D-galactose-induced rodent aging model, NOX2 expression was significantly upregulated in the ventral cochlear nucleus. NOX2-dependent oxidative stress may contribute to mitochondrial dysfunction and activate caspase-3-dependent apoptotic pathways within the central auditory system during aging (Du et al., 2015). Moreover, recent findings suggest that NOX2 mediates the selective vulnerability of OHCs in high-frequency regions, further supporting its potential as a therapeutic target for sensorineural hearing loss and offering new insights into the pathogenesis of ARHL (Qi M. et al., 2025). Similarly, NOX4 overexpression in transgenic mice has been shown to increase susceptibility to noise-induced hearing loss (Morioka et al., 2018). Collectively, these findings highlight the distinct spatial distribution and functional roles of individual NOX isoforms, emphasizing the importance of isoform-specific targeting strategies in future therapeutic approaches for ARHL.

Beyond NOX enzymes, mitochondrial dysfunction itself is a major source of ROS in the aging cochlea. When mitochondria are impaired, excessive ROS production accelerates degeneration of cochlear structures. For example, Cdk5rap1 knockout mice exhibit early-onset ARHL, with cochlear impairment linked to mitochondrial damage and elevated oxidative stress in the spiral ligament and stria vascularis (Miwa et al., 2023). Oxidative stress induces apoptosis in both cochlear hair cells and SGNs. This cell death contributes significantly to the progression of ARHL. Dysregulation of the Nrf2 antioxidant pathway has been shown to result in mitochondrial oxidative damage and activate caspase-3-dependent apoptosis in hair cells (Li et al., 2023). Oxidative stress can also initiate inflammatory responses that worsen cochlear injury. For instance, in SESN2-deficient mice, accelerated ARHL progression is associated with activation of the NLRP3 inflammasome, linking oxidative stress to inflammation-mediated hearing decline (Shi et al., 2017). Additionally, chronic diseases such as diabetes, hypertension, and hypercholesterolemia are closely associated with increased oxidative stress and mitochondrial dysfunction, both of which heighten the risk of ARHL (Tang et al., 2023). Moreover, unhealthy lifestyle habits–such as poor diet and lack of exercise–further exacerbate oxidative damage, thereby accelerating cochlear aging (Gopinath et al., 2011; Du et al., 2012; Tang et al., 2023). Undoubtedly, oxidative stress plays a central role in ARHL pathogenesis, driving mitochondrial dysfunction, apoptosis, inflammation, and epigenetic dysregulation. Targeting oxidative stress pathways offers a promising therapeutic strategy for ARHL.

### 3.4 Excessive autophagy

Autophagy is a fundamental cellular process responsible for the degradation and recycling of damaged organelles and misfolded proteins, thereby maintaining intracellular homeostasis and facilitating stress responses. Dysregulation of autophagy has been closely linked to various age-related diseases, including ARHL. One of the key mechanisms through which autophagy disorders contribute to ARHL is by promoting cell death. Notably, studies have revealed a significant upregulation of miR-34a in the aging cochlea, which is associated with impaired autophagic flux (Xiong et al., 2015; Pang et al., 2017). Overexpression of miR-34a disrupts the fusion of autophagosomes with lysosomes and then leads to the accumulation of autophagic vacuoles, ultimately triggering cell death and accelerating the onset of ARHL (Pang et al., 2017). In addition, autophagy dysfunction can contribute to ARHL through gene mutation-related pathways. For instance, mutations in the Cx26 gene have been shown to cause oxidative stress and impair autophagic activity, resulting in accelerated degeneration of cochlear cells. Mice with partial loss of Cx26 exhibit more rapid progression of ARHL, highlighting the gene’s critical role in maintaining cochlear integrity (Fetoni et al., 2018). Mitochondrial autophagy, or mitophagy, also plays an essential role in hearing preservation. Age-related decline in mitophagy has been observed in cochlear cells, which exacerbates hearing impairment (Oh et al., 2020). Specifically, mitophagy mediated by the PINK1/Parkin pathway becomes increasingly compromised with age and is associated with the aging of the central auditory system, including the auditory cortex and inferior colliculus (Youn et al., 2020). Interestingly, overexpression of SIRT1 has been shown to enhance the expression of PINK1 and Parkin in cochlear hair cells of aged mice, thereby mitigating hair cell loss and delaying the progression of ARHL (Xiong et al., 2019). Collectively, these findings underscore the critical role of autophagy dysfunction in the pathogenesis of ARHL. Targeting autophagy-related pathways through pharmacological activation, gene therapy, or combination treatments holds promising therapeutic potential for preventing or alleviating ARHL.

### 3.5 Hormonal modulation

The endocrine system plays a vital role in regulating physiological processes and maintaining homeostasis through hormone production. Age-related hormonal changes can disrupt this balance, contributing to age-related disorders–including ARHL (van den Beld et al., 2018; Frisina et al., 2021). Among hormonal influences, estrogen has received particular attention due to its potential protective effects on auditory function. A cross-sectional study involving 2,349 individuals highlighted that prolonged estrogen exposure, particularly early in life, is associated with a reduced risk of ARHL (Nam et al., 2024). Supporting this, a recent exome sequencing study identified sex-specific genetic associations with adult hearing impairment, providing direct evidence for gender-related hormonal regulation in ARHL pathogenesis (Lewis et al., 2022). Estrogen acts via estrogen receptors (ER-α and ER-β), which are expressed in key structures of the inner ear–including Reissner’s membrane, stria vascularis, spiral ligaments, spiral and vestibular ganglia, and hair cells–as well as central auditory regions such as the auditory cortex, cochlear nucleus, and inferior colliculus (Charitidi and Canlon, 2010; Milon et al., 2018; Shuster et al., 2019). There is a significant decline in ER-α expression in specific auditory cortical regions of aged mice, with ER-β levels remaining stable (Charitidi and Canlon, 2010). The cochlear localization of these receptors is crucial, as they influence hearing sensitivity, amplitude, and latency (Williamson et al., 2019). ER-β knockout mice showed extensive damage in the basal region of the organ of Corti, including hair cell loss and reduced spiral ganglion neurons, leading to profound deafness by 1 year of age (Simonoska et al., 2009). ER-α has also been linked to sex-based differences in ARHL, with higher expression observed in young female CBA/CAJ mice than in males (Motohashi et al., 2010). One mechanism for estrogen’s protective effects may involve the insulin-like growth factor-1 (IGF-1) pathway (Rodríguez-de la Rosa et al., 2017). IGF-1 serves as a neuroprotective agent critical for maintaining cellular functions such as growth activation, metabolism, proliferation, and differentiation, but its circulating levels decline with age. Several studies have highlighted the potential involvement of IGF-1 in hearing impairment, including ARHL (Iwai et al., 2006; Yamahara et al., 2015; Muus et al., 2017). IGF-1 knockout mice experienced significant hearing loss by 3 months, with severe waveform abnormalities and accelerated neural and vascular damage by 1 year (Riquelme et al., 2010). IGF-1’s decline with age and its protective role in cochlear health make it a promising therapeutic target. Preclinical studies have shown IGF-1 protects against hearing loss caused by noise or ototoxic drugs (Yamahara et al., 2015). Notably, in a clinical study of 11 patients with Laron syndrome, early IGF-1 therapy prevented hearing loss (Nakagawa et al., 2014).

Auditory development is highly sensitive to thyroid hormone. Hearing loss has been linked to conditions such as endemic iodine deficiency, resistance to thyroid hormone, and congenital hypothyroidism (Lichtenberger-Geslin et al., 2013). A deficiency of thyroid hormone during critical periods of inner ear development results in impaired pruning and long-term homeostatic maintenance of afferent synapses (Sundaresan et al., 2016). Song et al. (2008) reported abnormal turning curve and two-tone suppression in thyrotropin receptor mutant mice with hypothyroidism, in contrast to mice with normal cochlear development. Knockout of thyroid hormone transporters has been shown to cause delays in cochlear development, characterized by the loss of hair cells, supporting cells, and diminished cochlear potential (Sharlin et al., 2018). Beyond its essential role in development, thyroid hormone has also been implicated in the progression of ARHL. In particular, thyroid hormone receptor β1 has been shown to play a key role in maintaining auditory function and promoting cochlear hair cell survival in aging mice (Ng et al., 2015). These findings support the therapeutic potential of hormone-based and related interventions for preventing or treating the progression of ARHL.

### 3.6 Cellular signaling dysregulation

**FIGURE 2:**
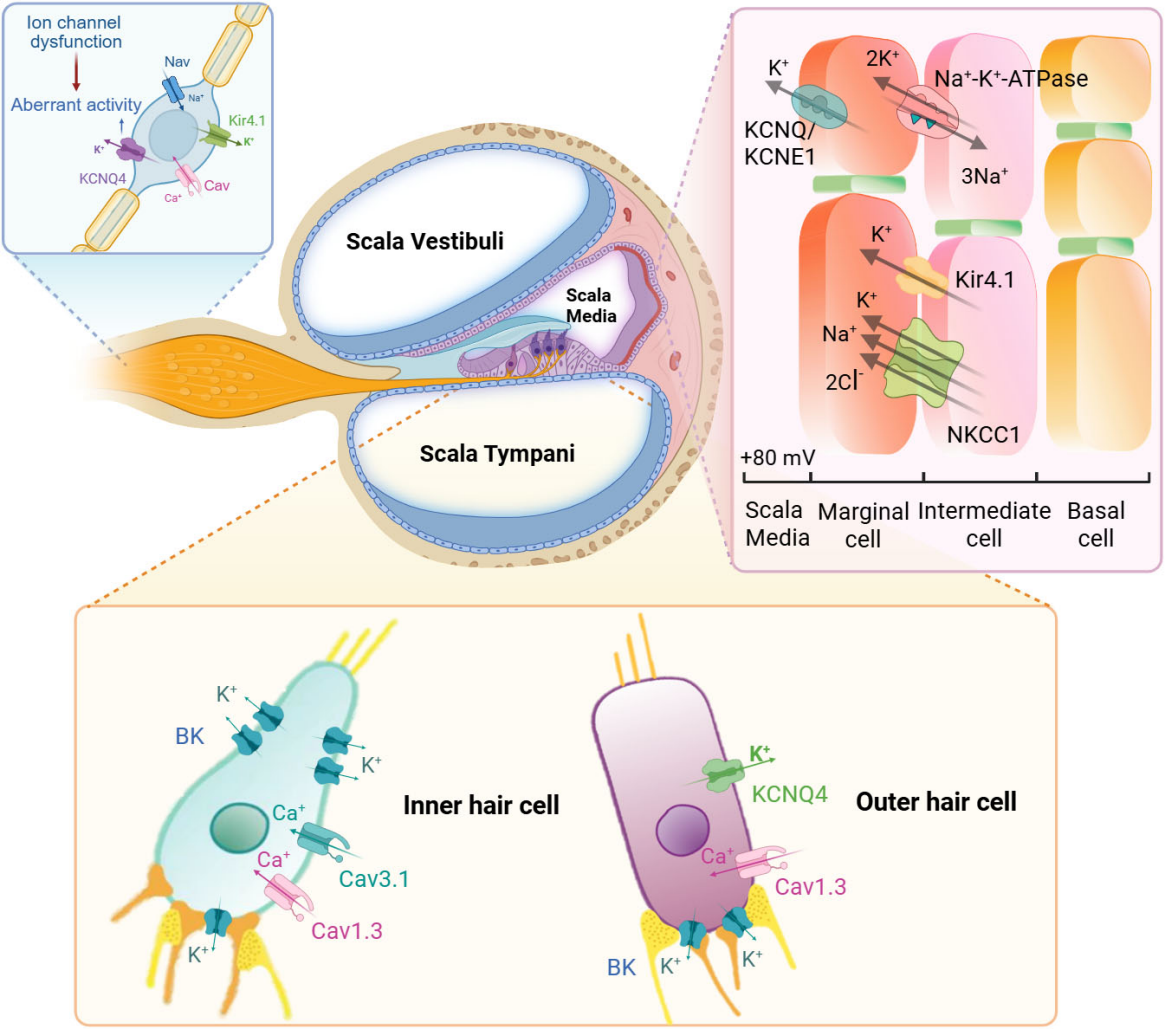
Ion channel and transporter dysfunctions in the pathogenesis of ARHL. Essential ion channels and transporters–including Na^+^/K^+^-ATPase, KCNQ/KCNE1 complexes, Kir4.1, and NKCC1–play critical roles in maintaining electrochemical gradients within cochlear cells. Inner hair cells primarily depend on Cav1.3 and Cav3.1 calcium channels, while outer hair cells utilize KCNQ4, Cav1.3, and BK channels to regulate calcium influx and support their unique electrophysiological functions. Ion channel dysfunction within the spiral ganglion disrupts neuronal activity, contributing to the neural components of ARHL pathophysiology. Created with Biorender.com.

Membrane ion channels and transport proteins are fundamental to auditory signal transduction and processing. With aging, dysfunction or degradation of these proteins can impair ion homeostasis and neural signaling within the cochlea, thereby contributing to ARHL. Disruption in the regulation of these cellular components compromises auditory sensitivity and signal processing, ultimately leading to reduced hearing function (Figure 2).

#### 3.6.1 Sodium-potassium-chloride cotransporter

The sodium-potassium-chloride cotransporter (Na^+^-K^+^-2Cl^–^, or NKCC) plays a crucial role in maintaining osmotic balance and ion concentration within animal cells by facilitating the electroneutral transport of Na^+^, K^+^, and Cl^–^ across the plasma membrane. NKCC1 is highly expressed in the marginal cells of the stria vascularis in the inner ear, where it is essential for maintaining the endocochlear potential (EP) through regulation of K^+^ concentration in the scala media–an essential condition for normal cochlear function and auditory transduction. Disruption or genetic deletion of NKCC1 in mice results in deafness and structural abnormalities in the inner ear, underscoring its critical role in auditory physiology (Delpire et al., 1999; Dixon et al., 1999). In ARHL models such as DBA and C57BL6 mice, a consistent decline in NKCC1 expression has been observed, which correlates with progressive structural degeneration and functional deterioration of the inner ear (Liu et al., 2014; Halonen et al., 2016).

Further evidence of NKCC1’s importance comes from pharmacological studies: administration of furosemide, an NKCC1 inhibitor, leads to a marked reduction in EP and increased auditory thresholds. Notably, furosemide-treated gerbils exhibit a temporal and frequency-specific pattern of hearing loss that closely mirrors the clinical progression of human presbycusis (Sewell, 1984; Schmiedt et al., 2002; Mills, 2006). These findings highlight the central role of NKCC1 in cochlear ion homeostasis and its involvement in ARHL pathogenesis. As such, NKCC1 represents a promising therapeutic target for the development of novel interventions aimed at preventing or mitigating age-related auditory decline.

#### 3.6.2 Na^+^/K^+^-ATPase

Sodium-potassium adenosine triphosphate (Na^+^/K^+^-ATPase), also known as the Na^+^-K^+^ pump, is an essential membrane-bound enzyme that actively transports sodium ions out of the cell and potassium ions into the cell against their respective concentration gradients. In the cochlea, Na^+^/K^+^-ATPase is expressed in the lateral wall, specifically within stria vascularis, where it plays a critical role in maintaining the EP (Adachi et al., 2013). Age-related degeneration of the cochlea involves progressive atrophy of the stria vascularis and a concomitant decline in Na^+^/K^+^-ATPase expression. Studies using Mongolian gerbil models have demonstrated a marked reduction in immunoreactive Na^+^/K^+^-ATPase in both apical and basal cochlear regions by 20 months of age, with the regions lacking immunostaining expanding progressively over time (Schulte and Schmiedt, 1992). At the molecular level, aging is associated with coordinated downregulation of multiple subunits of Na^+^/K^+^-ATPase. In CBA/CaJ mice, expression levels of the α1, β1, and β2 subunits decline significantly at both the gene and protein levels. Notably, the predominant α1-β1 heterodimer, abundant in young adult cochleae, becomes largely undetectable in aged animals (Ding et al., 2018). These molecular and histological changes correspond closely with functional impairments, as reduced Na^+^/K^+^-ATPase activity correlates with decreased endolymphatic potassium concentration and EP reduction (Lippincott and Rarey, 1997). Additionally, the enzyme’s involvement extends to acquired forms of hearing loss. Aminoglycoside antibiotics exert ototoxic effects partly through inhibition of Na^+^/K^+^-ATPase. While the initial damage may be reversible, prolonged exposure often leads to irreversible cochlear injury and permanent hearing loss in approximately 63% of affected individuals (Xie et al., 2011; Ganesan et al., 2018), highlighting the enzyme’s pivotal role in both intrinsic and extrinsic hearing loss pathways. These findings underscore the central role of Na^+^/K^+^-ATPase in both age-related and drug-induced auditory dysfunction.

#### 3.6.3 Potassium channels

Kv7.4, encoded by KCNQ4 gene, is a member of the Kv7 family of voltage-gated potassium channels and is critically expressed at the basal pole of cochlear OHCs. It mediates K^+^ efflux and constitutes the predominant K^+^ conductance current of OHCs, known as I_*K,n*_ (Marcotti and Kros, 1999). The essential role of Kv7.4 channel in maintaining OHC function and survival has been demonstrated through genetic studies. Kcnq4 knockout mice, as well as humans with loss-of-function mutations in Kcnq4, exhibit progressive hearing loss accompanied by slow degeneration of OHCs (Kharkovets et al., 2006; Van Eyken et al., 2006). The absence of Kv7.4 disrupts K^+^ homeostasis, resulting in chronic depolarization of OHCs. This depolarized state may lead to excessive Ca^2+^ influx via voltage-gated Ca^2+^ channels, contributing to cellular stress and eventual OHC degeneration (Rüttiger et al., 2004). Notably, pharmacological activation of Kv7.4 has been identified as a feasible approach to preserve auditory function and prevent OHC loss in models of ARHL (Rim et al., 2021; Peixoto Pinheiro et al., 2022). In addition, Kv7.1, encoded by the KCNQ1 gene, plays a role in auditory physiology through its expression on the apical membrane of marginal cells in the stria vascularis of the cochlear lateral wall. Studies have shown that hearing loss in LIMP2-deficient mice is associated with early depletion of the KCNQ1/KCNE1 channel complex in these cells (Knipper et al., 2006). However, the specific role of KCNQ1 in the pathogenesis of ARHL remains to be elucidated.

Kir4.1, encoded by the KCNJ10 gene, is an inwardly rectifying potassium channel predominantly expressed in the inner ear of mammals. It is primarily localized in the cochlear lateral wall, SGNs, and supporting cells within the organ of Corti. Kir4.1 plays a critical role in development and maintenance of the cochlear EP, which is essential for normal inner ear function and sound transduction (Chen and Zhao, 2014). KCNJ10 knockout mice exhibit profound deafness, characterized by a loss of EP, reduced acoustic startle responses, and significant degeneration of various inner ear structures (Marcus et al., 2002; Rozengurt et al., 2003). Notably, recent studies have reported age-dependent alterations in Kir4.1 expression in neural crest-derived cells of the mouse and human cochlea (Liu et al., 2019). Further research is necessary to elucidate the role of Kir channels in ARHL.

Large-conductance Ca^2+^ and voltage-activated K^+^ (BK) channels expressed in cochlear hair cells are crucial for mediating fast-activating outward K^+^ currents, which are essential for high-frequency hearing in mammals (Latorre et al., 2017). Mice lacking the BKα subunit exhibit progressive hearing loss (Rüttiger et al., 2004; Pyott et al., 2007). Notably, both the protein and the mRNA expression levels of BK channels in the cochlea decline significantly and progressively with age (Pan et al., 2016). Consistently, BK currents in IHCs have also been shown to decrease with age (Jeng et al., 2021). Given the pivotal role of BK channels in hearing function, it is plausible that contribute to ARHL. However, direct evidence linking BK channel dysfunction to ARHL remains limited. Further investigations are necessary to clarify the potential involvement of BK channels in the pathogenesis of ARHL.

#### 3.6.4 Calcium channels

Cav1 channels mediate L-type Ca^2+^ currents that are essential for triggering the exocytotic release of glutamate at the specialized ribbon synapses of cochlear IHCs. Genetic studies in animal models have established Cav1.3 channels as the predominant Cav channel subtype in IHCs, playing a critical role in auditory signal transmission (Platzer et al., 2000; Brandt et al., 2003). Recent research indicates that interleukin-6-induced upregulation of Cav1.3 in IHCs may be a key contributing factor for ARHL. Pharmacological inhibition of Cav1.3 channels has been shown to attenuate cochlea damage and rescued hearing in ARHL models (Lu et al., 2024). Cav3 calcium channels, also known as T-type calcium channels, include three subtypes: Cav3.1, Cav3.2, and Cav3.3. These channels are crucial for regulating cytosolic Ca^2+^ concentrations in the cochlea. In mouse models of ARHL, upregulation of Cav3 channels in SGNs has been observed and may promote SGN degeneration through activation of the calpain-2-AIF apoptotic pathway (Geng et al., 2021). Despite these findings, the detailed molecular mechanisms underlying the involvement of calcium channels in ARHL, as well as their therapeutic potential, need further investigation and supporting evidence.

### 3.7 Osteoporosis

Osteoporosis is a significant age-related disorder characterized by reduced bone mass and increased bone fragility, which heightens the risk for fractures (Delmas, 2002). The ossicles in the middle ear, small bony structures, play a crucial role in transmitting sound energy from the outer ear to the inner ear. Osteoporosis may contribute to ARHL by compromising the integrity and functionality of these middle ear structures. Several cohort and longitudinal studies have demonstrated an increased risk of ARHL associated with osteoporosis (Kahveci et al., 2014; Yeh et al., 2015; Kim et al., 2018; Yoo et al., 2020). The mechanisms underlying hearing loss in osteoporosis are complex and not fully understood. One possible explanation is that decreased bone mineral density leads to microfractures in the ossicles. Additionally, secondary bone remodeling could alter the normal sound transmission properties of the middle ear, manifesting as a decrease in middle ear resonance frequency and an increase in static compliance (Baytaroglu et al., 2021). In addition, imbalance in bone formation and bone resorption from osteoporosis may play an important role in dysfunctional ionic metabolism leading to sensory neural hearing loss. Abnormal increase in bone resorption can elevate calcium concentrations in the endolymph, interfering with endolymph potential (Upala et al., 2017; Xu et al., 2021). Osteoprotegerin, a key regulator of bone remodeling, plays a significant role in this context. Temporal bones in osteoprotegerin knockout mice exhibit abnormal bone remodeling, demyelination, and degeneration of the cochlear nerve. Osteoprotegerin deficiency also activates extracellular signal-regulated protein kinases, sensitizing spiral ganglion cells to apoptosis (Kao et al., 2013). Moreover, low vitamin D levels have been associated with hearing loss in the elderly. It has been speculated that vitamin D deficiency may contribute to hearing loss through its role in calcium absorption and bone metabolism (Bener et al., 2018). However, this hypothesis remains uncertain due to insufficient data supporting the specific mechanism involved.

## 4 ARHL promotes the occurrence and progression of other diseases

**FIGURE 3:**
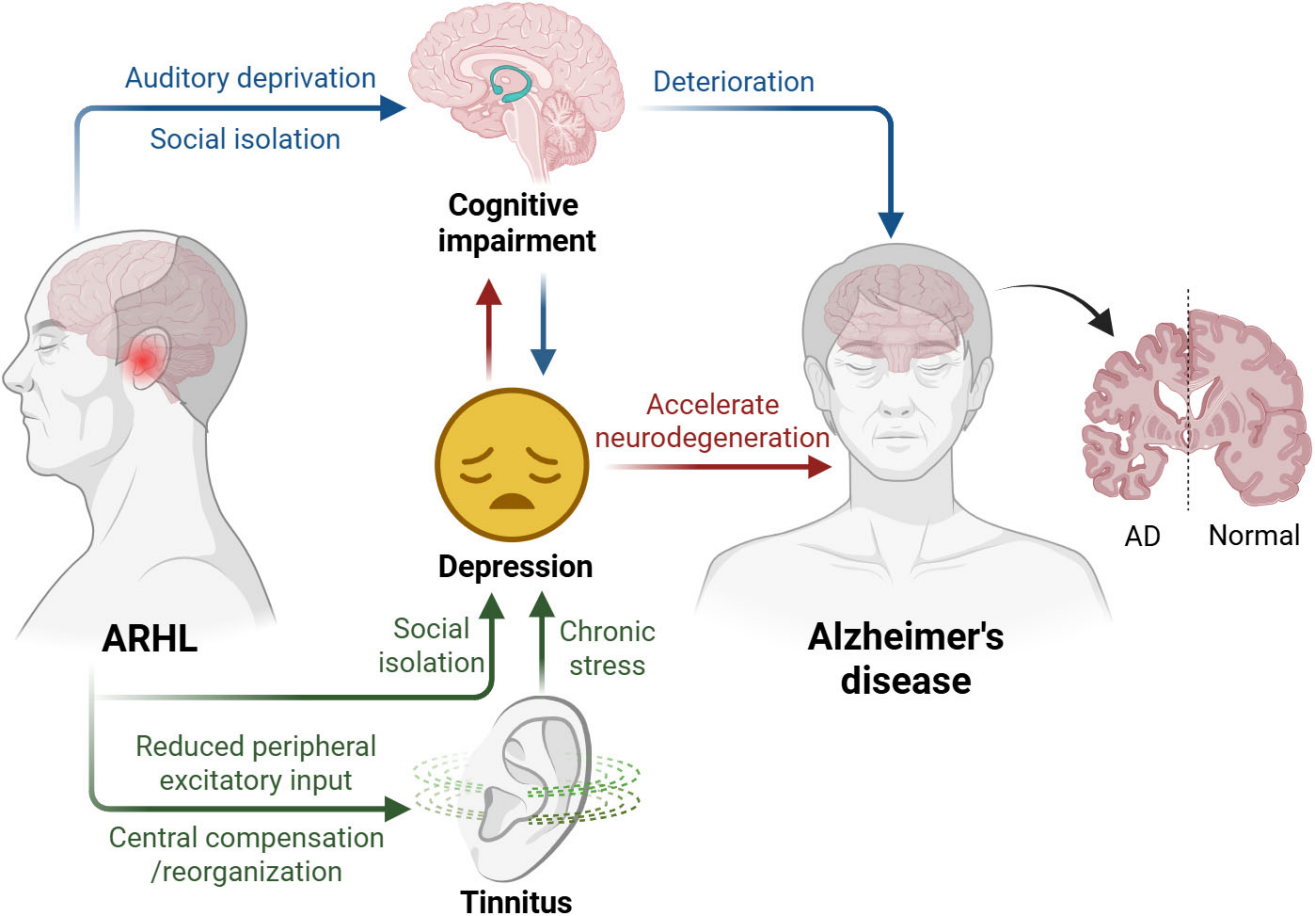
Emotional and cognitive disorders caused by ARHL. ARHL contributes to auditory deprivation and social isolation, which in turn promote cognitive decline and increase the risk of depression. Reduced peripheral auditory input induces central neural compensation and reorganization, potentially leading to tinnitus. Tinnitus, along with chronic stress and social withdrawal, further contributes to emotional distress. Depression and associated stress responses may accelerate neurodegenerative processes, thereby exacerbating the onset and progression of Alzheimer’s disease (AD). Created with Biorender.com.

Age-related hearing loss is increasingly recognized not merely as a peripheral sensory deficit, but as a multifactorial condition that interacts with and exacerbates a range of age-associated diseases. Emerging evidence suggests that ARHL actively contributes to the onset and progression of several comorbidities, including tinnitus, depressive disorders, cognitive impairment, and Alzheimer’s disease (Figure 3). These associations underscore the systemic nature of ARHL and highlight the need for early detection and intervention to prevent downstream pathological consequences.

### 4.1 Tinnitus

The incidence of hearing loss, tinnitus, and hyperacusis increases with age, and their interrelationships have been the subject of extensive research (Schecklmann et al., 2014; Aazh et al., 2017). Although the prevalence of tinnitus does not always directly correct with the degree of hearing loss in the standard audiometry frequency range, chronic tinnitus is frequently accompanied by some degree of hearing impairment (Eggermont, 2017). Age-related hearing loss reduces excitatory input from the auditory periphery, which leads to maladaptive central auditory system compensation–a key mechanism believed to underlie the development of tinnitus in older. Histopathological studies in elderly tinnitus patients have revealed that strial vascular atrophy and outer hair cell loss are more commonly observed in individuals with tinnitus than in those without it (Terao et al., 2011). Moreover, aging is increasingly recognized not merely as a contributor to hearing loss, but as an independent risk factor for tinnitus itself (Reisinger et al., 2023). This suggests that the aging process may lead to structural and functional changes in the auditory system that predispose individuals to both ARHL and tinnitus, reinforcing their frequent coexistence in older adults.

### 4.2 Depression

The relationship between ARHL and late-life depression is well documented. Older adults with hearing loss tend to experience higher levels of depression and lower scores on depression scales compared to those with normal hearing (Brewster et al., 2018). Several theories attempt to explain the connection between hearing loss and late-life depression. Individuals with hearing difficulties often struggle to follow conversations, which can lead to frustration in interactions with family and friends, diminishing their enjoyment of social activities and relationships. Additionally, hearing loss can make it challenging for individuals to engage in activities, primarily due to discomfort lacking auditory input. These factors may contribute to the development of depressive symptoms. Research has shown that individuals with severe hearing loss report lower levels of physical activity and reduced engagement in physical activities (Chen et al., 2014). Interestingly, one study identified a linear relationship between the degree of depression and the severity of hearing loss (Cacciatore et al., 1999), while another found that the pure-tone audiometry hearing threshold was linked to depression, but not to self-reported hearing loss (Lee et al., 2010). The timely use of hearing aids in patients with age-related hearing loss helps alleviate depressive symptoms and reduce the likelihood of developing depression.

### 4.3 Cognitive impairment

Emerging evidence highlights a significant association between ARHL and cognitive decline. Several mechanistic hypotheses have been proposed to explain this relationship, with the common cause hypothesis, cascade hypothesis, and cognitive load hypothesis being particularly salient in disease development. Each hypothesis appears to exert distinct effects depending on the individuals (Stahl, 2017). The association between hearing loss, cognitive decline, and dementia has gained considerable attention, especially following the 2020 Lancet Commission report (Livingston et al., 2020). Although further research is needed, current evident suggests that hearing loss may lead to auditory deprivation, contributing to functional and structural impairments in brain, including accelerated cognitive decline (Jafari et al., 2019) and reduced temporal lobe volume compared to individuals with normal hearing (Armstrong et al., 2019). Notably, auditory rehabilitation–such as cochlear implants or hearing aids–has been associated with improved cognitive performance, implying that hearing restoration interventions could serve as a potential strategy to mitigate age-related cognitive impairment (Castiglione et al., 2016).

### 4.4 Alzheimer’s disease

Alzheimer’s disease (AD) is the most prevalent cause of dementia, accounting for 60%–70% of all dementia cases. According to the World Report, an estimated 35.6 million people worldwide were living with AD and related dementias in 2010, a number projected to rise to 66 million by 2030 and 115 million by 2050 (Wortmann, 2012). Beyond cognitive dysfunction, AD is frequently associated with comorbid conditions, including cardiovascular diseases, malignancies, and sensory impairments (e.g., vision and hearing loss) (Masters et al., 2015). Given that both AD and hearing loss progress with aging, emerging evidence suggests that hearing impairment may exacerbate cognitive deterioration in AD patients. Although hearing aid use has not been conclusively shown to reduce dementia risk, studies indicate that hearing loss exceeding 25 dB is associated with a higher incidence of dementia (Lin et al., 2011). Notably, AD exhibits a strong link with auditory pathway dysfunction. Patients often demonstrate impaired central auditory processing, and neuropathological changes have been identified in the auditory system (Idrizbegovic et al., 2011). Neuroimaging studies reveal cortical atrophy in AD patients, characterized by sulcal widening, gyral thinning, and reduced brain volume. Furthermore, both AD mouse models and human patients display elevated hearing thresholds, suggesting that hearing deficits may contribute to AD (Uhlmann et al., 1989; Goll et al., 2011; O’Leary et al., 2017). These findings underscore the need for early intervention strategies, such as hearing restoration, cognitive training, lifestyle modification, and pharmacological therapies, which may slow cognitive decline, improve quality of life, and delay the onset or progression of dementia-related pathology.

## 5 Intervention and treatment strategies

### 5.1 Hearing aids and cochlear implants

Clinical interventions for ARHL are currently limited primarily to hearing aids and cochlear implants, as safe, reliable, and effective biological therapies remain unavailable. Hearing aids are widely used as non-medical rehabilitation tools for individuals of all ages with partial hearing loss (mild to moderate hearing loss). Their primary function is to amplify external sounds, compensating for the patient’s reduced hearing sensitivity. This not only improves overall hearing ability but also enhances auditory-verbal communication (GBD 2019 Hearing Loss Collaborators, 2021). A meta-analysis of five randomized controlled trials involving 825 patients aged 69–83 years with ARHL demonstrated that hearing aid interventions effectively improve hearing health and quality of daily life (Brennan-Jones et al., 2018). A survey of 2,040 individuals over the age of 50 with hearing loss revealed a positive correlation between hearing aid use and cognitive function scores, indicating that hearing aids can slow the rate of cognitive decline in later life (Maharani et al., 2018). Unfortunately, hearing aids can enhance the perception of weak sounds but are insufficient to fully restore normal auditory perception, particularly offering only limited benefits for understanding of speech in noisy environments (Nieman et al., 2016; Lesica, 2018). It is crucial to continue advancing the development of devices capable of restoring normal auditory perception. Moreover, hearing aids are powerless against severe hearing loss.

Cochlear implantation is currently the most direct and effective rehabilitation method for addressing severe to profound hearing loss. A prospective cohort study of 70 patients aged 65 and older with profound ARHL showed that within 1–7 years after cochlear implantation, over 80% of patients experienced sustained improvements in speech perception scores and quality of life, along with enhanced cognitive function (Mosnier et al., 2018). Two recent studies have also confirmed this, showing that cochlear implantation can improve cognitive function in patients with profound ARHL (Völter et al., 2022; Völter et al., 2023). The effectiveness of cochlear implantation is limited by the integrity of the auditory nerve and the condition of the hair cells. The integrity of the auditory nerve is the main factor influencing the outcome of cochlear implantation, which is why only some ARHL patients benefit, and the restoration of hearing is limited. Both hearing aids and cochlear implants cannot fundamentally improve the pathological condition of hearing loss, and their effectiveness varies from person to person. Therefore, research into the mechanisms of hearing loss and targeted biological therapies is urgently needed.

### 5.2 Pharmacological interventions

#### 5.2.1 Cholesterol-lowering drugs

Excessive cholesterol can accumulate along the walls of blood vessels, leading to the formation of fibrotic tissue and atherosclerotic plaques, which may eventually cause vascular obstruction and various related diseases. In older adults, a decline in metabolic capacity further impairs cholesterol clearance, increasing the risk of these complications. A retrospective longitudinal study involving 1,978 individuals found that the age-related increase in hearing thresholds was significantly lower in individuals with low-density lipoprotein (LDL) cholesterol levels below 100 mg/dL compared to those with LDL levels above 100 mg/dL (Kim et al., 2019). These findings suggest that strict management of LDL cholesterol–through dietary regulation and pharmacological interventions such as statins and omega-3 fatty acids–may positively influence ARHL (Gopinath et al., 2010; Gopinath et al., 2011; Rahimi et al., 2024). Supporting evidence from animal studies has demonstrated that long-term systemic administration of atorvastatin, a type of statin, can delay the onset of ARHL and mitigate cochlear pathological changes in C57BL/6J mice (Syka et al., 2007; Choo et al., 2022). It is worth noting that current studies on statins and hearing are limited and yield inconsistent results due to methodological differences and lack of prospective trials. Statins may protect hearing through cholesterol-dependent and independent mechanisms, but optimal drug type, dosage, and treatment duration remain unclear (Whitlon, 2022). Additionally, their ability to cross the blood-labyrinth barrier and potential central nervous system effects warrant further investigation. Importantly, side effects such as muscle pain and cognitive issues limit long-term use, highlighting the need for personalized treatment and rigorous clinical evaluation.

#### 5.2.2 Cochlear vasodilators

Maintaining adequate cochlear blood supply is essential for preserving the endocochlear potential and endolymph production–key components for normal auditory function. Impaired cochlear perfusion has been identified as a significant contributor to sensorineural hearing loss (Shi, 2011). Pharmacological agents that enhance cochlear circulation, such as vasodilators and vasoactive compounds, show promise as potential therapeutic strategies. For example, hydrogen sulfide, a known vasodilator, has been shown to protect the inner ear from noise-induced hearing loss by reducing cochlear damage and preserving auditory function (Li et al., 2011). Magnesium has also demonstrated notable otoprotective properties, enhancing cochlear blood flow, modulating glutamate receptor activity, and regulating calcium channel permeability (Haupt and Scheibe, 2002; Alvarado et al., 2015). When administered in combination with natural antioxidants or vitamins, magnesium may exert synergistic effects, offering an effective therapeutic approach to ARHL (Alvarado et al., 2015; Alvarado et al., 2018). However, vasodilators, though promising for improving cochlear blood flow, present several limitations and potential side effects. These include limited efficacy with significant interindividual variability, inability to reverse structural damage in the cochlea, potential systemic side effects, issues with drug tolerance and metabolic clearance, and a lack of robust large-scale clinical evidence. Therefore, while vasodilators may serve as adjunctive treatments for ARHL, their therapeutic value must be carefully weighed against these limitations and risks. Future efforts should focus on precise patient stratification and individualized treatment approaches, supported by more comprehensive clinical research, to optimize both the efficacy and safety of vasodilator-based therapies.

#### 5.2.3 Rapamycin

Rapamycin, an inhibitor of the mechanistic target of rapamycin (mTOR), is widely recognized for its immunosuppressive properties and its influence on various signaling pathways, including those regulating metabolism, cell proliferation, immune responses, and survival. It has been proposed as a potential therapeutic agent for numerous age-related diseases (Lee et al., 2024). The mTOR signaling complexes, mTORC1 and mTORC2, play distinct roles in maintain cochlear hair cell function. While mTORC1 has a detrimental effect on hair cell survival, it also contributes positively to hair cell regeneration, necessitating precise modulation for therapeutic applications. In contrast, mTORC2 appears to support hair cell survival and protection (Cortada et al., 2021). Overactivation of mTORC1 has been identified as a key factor in ARHL, suggesting that reducing its activity in cochlear hair cells could help prevent ARHL (Fu et al., 2018). Recent research highlights that activating mTORC2 could be a promising approach to preserve hair cell function and mitigate ARHL progression (Fu et al., 2022). The beneficial effects of rapamycin are largely attributed to its inhibition on mTORC1 activity. Studies have shown that dietary supplementation with rapamycin during late mid-life can reduce age-related loss of outer hair cells and delay ARHL in UM-HET Mice (Altschuler et al., 2018; Altschuler et al., 2021). However, rapamycin’s immunosuppressive side effects present a significant challenge for its clinical use. Further exploration of the mTOR pathway may lead to more effective and targeted strategies to prevent ARHL while minimizing adverse effects.

#### 5.2.4 Anti-inflammatory and antioxidant therapy

Metformin (N, N-dimethylbiguanide), a hypoglycemic agent originally derived from the herb *Galega officinalis* has demonstrated beneficial effects beyond glucose regulation. It has been shown to enhance both autophagy and mitochondrial function, thereby alleviating a newly characterized “inflammaging” profile–an age-associated chronic inflammation pattern resembling that observed in diabetes (Bharath et al., 2020). In auditory HEI-OC1 cells, metformin prevented H_2_O_2_-induced premature senescence by restoring mitophagy (Cho et al., 2025). Furthermore, in vivo studies have indicated that metformin significantly mitigates ARHL, cellular apoptosis, and neurodegeneration through modulation of the unfolded protein response and activation of the AMPK/ERK1/2 signaling pathways (Cai et al., 2020). L-ergothioneine, a naturally occurring amino acid, holds significant potential as a therapeutic agent, having demonstrated efficacy in various disease models, including neurological disorders. It is specifically transported into cells via the organic cation/ergothioneine transporter (OCTN1) receptor, where it functions as a potent antioxidant and anti-inflammatory molecule. Recent studies have shown that L-ergothioneine can mitigate ARHL by preventing key pathological features such as cochlear oxidative stress, apoptosis, and chronic inflammation (Bauer et al., 2024). Coenzyme Q10 (CoQ10), a major antioxidant from the ubiquinone family naturally synthesized in the body, stabilizes the mitochondrial membrane, thereby inhibiting mitochondrial depolarization and the release of cytochrome c. Oral supplementation with the mitochondrial antioxidants such as CoQ10 and α-lipoic acid has been shown to suppress Bak expression and reduce cell death in the cochlea, prevent ARHL in C57BL/6J mice (Someya et al., 2009). A randomized controlled clinical trial further demonstrated that, compared with vitamin E, CoQ10 treatment significantly improved pure tone audiometry thresholds at 1000, 2000, 4000, and 8000 Hz in patients with ARHL (Guastini et al., 2011). Moreover, a recent clinical trial reported that adding CoQ10 to the standard treatment regimen for patients with tinnitus associated with ARHL significantly reduced tinnitus-related disability, improved sleep disturbances, and decreased tinnitus loudness (Abbasi et al., 2025).

#### 5.2.5 Hormonotherapy

As outlined in the preceding sections, hormonal regulation plays a critical role in auditory development, cochlear homeostasis, and the pathogenesis of ARHL. Declining levels of key hormones such as estrogen, IGF-1, and thyroid hormone with age have been associated with increased vulnerability to hearing loss. These mechanistic insights provide a strong rationale for exploring hormone-based therapies as potential interventions to prevent or mitigate ARHL. Clinical studies have shown that postmenopausal women with higher serum aldosterone levels and those receiving hormone replacement therapy exhibit a lower incidence of ARHL (Price et al., 2009). Supporting this observation, experimental studies have demonstrated that aldosterone hormone treatment can help to prevent and restore cochlear function in the elderly (Halonen et al., 2016). In a separate study, the effects of estradiol on hearing were evaluated in middle-aged female CBA/CaJ mice (Williamson et al., 2019). These findings suggest that hormones, such as estradiol, have neuroprotective properties in the auditory system that may help prevent or delay certain forms of ARHL. However, not all hormone treatments are beneficial. A 4-months combined regimen of estrogen and progestins was found to accelerate ARHL by impairing OHC function and reducing overall auditory sensitivity, compared with estrogen monotherapy (Price et al., 2009). This highlights the potential limitations of hormone-based therapies and underscores the need for careful consideration of hormone combinations.

#### 5.2.6 Drugs that target ion channels/receptors

Emerging evidence increasingly supports the feasibility and therapeutic potential of targeting ion channels and receptors in the treatment of ARHL. Recent studies have identified several promising molecular targets that may offer effective intervention strategies. One particularly promising target is the Kv7.4 potassium channel. Pharmacological activation of Kv7.4 using ACOU085–a novel small molecule agonist–has been shown to significantly attenuate age-related auditory threshold shifts and to prevent OHC loss in the SAMP8 mouse model (Peixoto Pinheiro et al., 2022). These findings suggest that Kv7.4 activation may represent a viable therapeutic approach not only for ARHL but also for other hearing disorders involving Kv7.4 dysfunction. Another key target is the voltage-gated Kv3.1 channel, which is highly expressed in fast-spiking neurons throughout the auditory pathway. Oxidative stress-induced inhibition of Kv3.1 has been implicated in ARHL pathogenesis. Notably, melatonin has demonstrated potential protective effects by counteracting this mechanism, suggesting its possible utility in preserving auditory function (Spinelli et al., 2024). The α9α10 nicotinic acetylcholine receptor (nAChR) complex, located on OHCs, mediates the protective effects of the medial olivocochlear system. Enhancing MOC activity through modulation of these receptors may provide a novel strategy for ARHL prevention (Boero et al., 2020). Furthermore, the development of specific agonists for other ion channels, such as Kir4.1 and BK channels, broadens the therapeutic landscape. These advancements collectively underscore ion channels and receptors as compelling and multifaceted targets in the development of treatments for ARHL. However, targeting ion channels and receptors may also pose significant risks of off-target effects and adverse outcomes, particularly due to the widespread distribution and functional importance of these channels in other tissues and organ systems. These adverse effects can be attributed to several factors, including poor target selectivity, inappropriate dosing, individual variability in ion channel expression, and compensatory mechanisms activated in response to channel modulation. Therefore, ongoing research should aim to refine therapeutic selectivity, minimize systemic exposure, and optimize delivery to the auditory system.

#### 5.2.7 Traditional Chinese medicine

**FIGURE 4:**
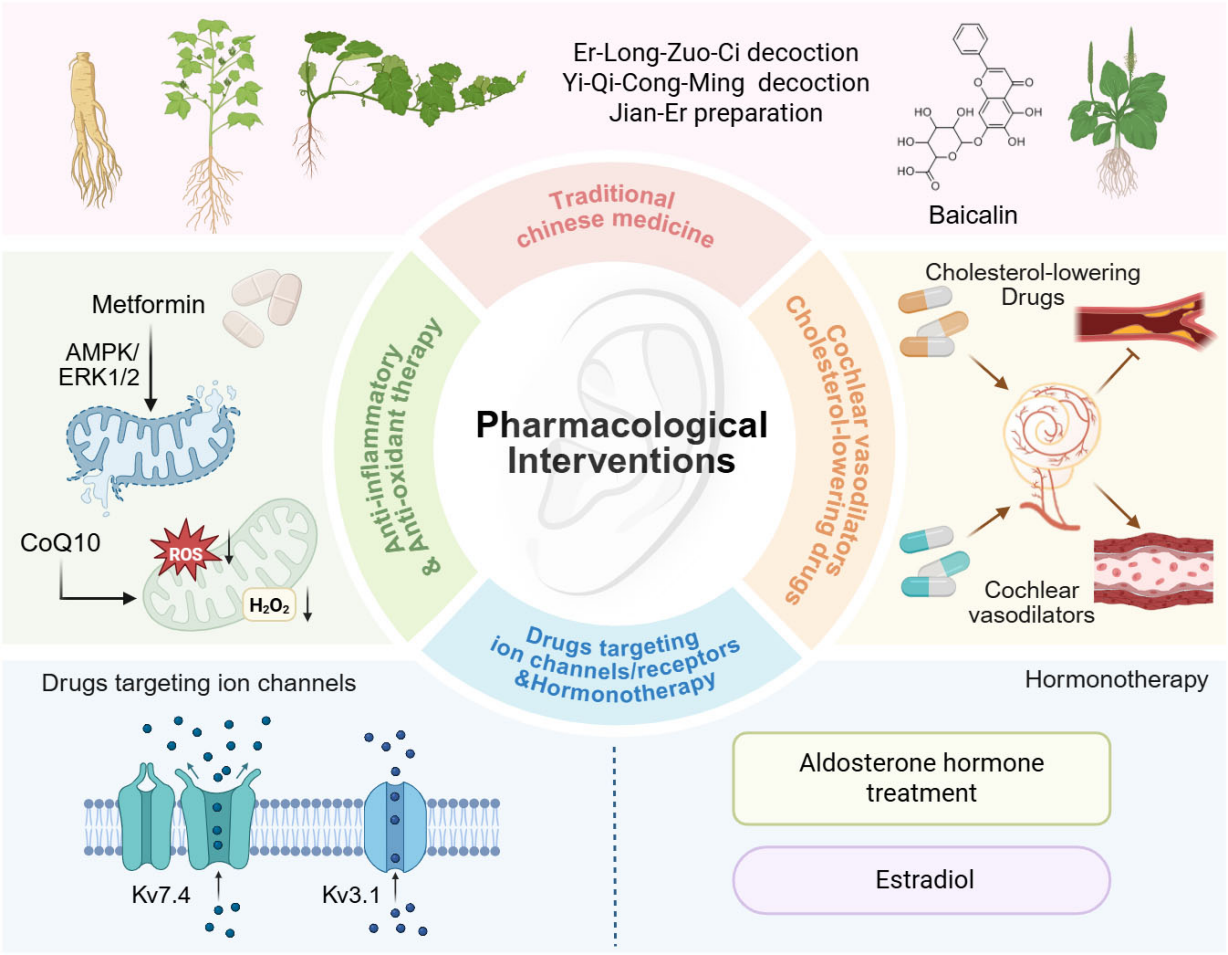
Current pharmacological strategies for managing ARHL. Traditional Chinese medicines, including Er-Long-Zuo-Ci decoction, Yi-Qi-Cong-Ming decoction, Jian-Er preparation, and Baicalin, have shown potential therapeutic effects. Metformin modulates the AMPK/ERK1/2 signaling pathways, while Coenzyme Q10 (CoQ10) reduces reactive oxygen species (ROS) to mitigate oxidative stress. Pharmacological modulation of ion channels, such as Kv7.4 and Kv3.1, along with hormone therapies like aldosterone and estradiol, offer targeted approaches. Cholesterol-lowering agents and cochlear vasodilators enhance cochlear blood flow, contributing to improved auditory function. Created with Biorender.com.

Traditional Chinese Medicine (TCM) has gained global recognition for its distinctive advantages, including a lower incidence of side effects, a wide range of therapeutic targets, and a deep-rooted historical background. Herbal formulations in TCM are based on the principles of syndrome differentiation and holistic regulation. This synergistic, multi-target approach not only enhances therapeutic efficacy but also minimizes adverse effects. Several well-established herbal formulas have demonstrated efficacy in managing ARHL, supported by both clinical and preclinical studies (Figure 4). One such formulation is the Er-Long-Zuo-Ci (ELZC) decoction, a classical kidney-tonifying prescription listed in the Chinese Pharmacopeia. Clinical observational studies suggest that ELZC may improve hearing function in elderly individuals without causing significant adverse effects (Dong et al., 2016; Liu et al., 2021). The protective effects of ELZC against ARHL are thought to involve multiple mechanisms, including attenuation of cellular senescence, modulation of inflammatory responses, and enhancement of synaptic connectivity. These effects are associated with key signaling pathways, notably the JNK/STAT3 and ERK cascades (Liu et al., 2021). Our recent study further identified baicalin–the primary active constituent of ELZC–as a critical agent in alleviating age-related cochlear damage and hearing loss in DBA/2J mice. Baicalin exerts its effects through multi-target and multi-pathway mechanisms, including anti-inflammatory actions, modulation of sex hormone-related pathways, and activation of potassium channels (Shi et al., 2024). Jian-Er Preparation (JEP) is a traditional herbal formulation with otoprotective properties, grounded in the TCM principles of regulating the liver and gallbladder. It has been shown to effectively delay the progression of ARHL by targeting mitochondrial dysfunction. Specifically, JEP attenuates mitochondrial DNA damage, inhibits the release of cytochrome C, and suppresses caspase-dependent apoptosis via the mitochondrial pathway. These protective actions contribute to the preservation of cochlear hair cells and SGNs, thereby reducing age-related auditory degeneration (Xuan et al., 2018). Yi-Qi-Cong-Ming (YQCM) Decoction is a classical *Bushen Yiqi* (tonify kidney and replenish Qi) prescription widely employed in Chinese society for centuries for preventing aging-related decline. Preclinical research reveals that YQCM can mitigate age-related reductions in cochlear hair cell populations, potentially through modulating the apoptotic process in hair cells (Yang et al., 2022).

In addition to ARHL, several herbal formulations, individual herbs, and active constituents have shown promising effects in treating sensorineural hearing loss caused by ototoxic drugs and noise exposure. However, their efficacy specifically for ARHL remain inconclusive (Wu et al., 2024). In summary, while TCM shows considerable potential in the treatment of ARHL and other auditory disorders, several challenges persist. These include a limited number of clinical trials–often with small sample sizes–lack of comprehensive and objective evaluations of adverse effects, suboptimal study designs, short follow-up durations, and significant variability in the quality and composition of herbal ingredients. To advance the scientific understanding and clinical application of TCM in ARHL, three key strategies are essential: Conduct large-scale, multicenter, double-blind, placebo-controlled randomized clinical trials to generate robust and generalizable evidence. Establish a comprehensive safety database to systematically assess the risk profiles, support regulatory oversight, and facilitate international recognition. Employ advanced analytical technologies, organoid-based platforms, and deep learning models to characterize and explore key bioactive constituents and underlying mechanisms.

### 5.3 Gene therapy and cell therapy

Advance in genomic research have identified multiple genes implicated in the pathogenesis of ARHL, such as DFNA5, MYO6, GRM7, GRHL2, KCNQ4, SLC26A4, and CDH23 (Ciorba et al., 2015; Bouzid et al., 2018). These genes can be broadly categorized into two functional groups: structural and functional genes of the inner ear and oxidative stress-related genes. These findings suggest that genetic modulation–either through gene silencing, upregulation, or editing–could serve as a viable therapeutic strategy for ARHL. The first successful attempt to apply a gene therapy strategy for treating deafness involved researchers using a recombinant adeno-associated virus (rAAV) to deliver the VGLUT3 gene to hair cells in VGLUT3 mutant mice, restoring hearing in the Vglut3 model mice (Akil et al., 2012). Following this, AAV-mediated gene therapy strategies rapidly advanced in the treatment of hereditary deafness, such as target genes OTOF^–/–^, TMC1^Y182C/Y182C^, and KCNQ4^W276S/+^. Recent groundbreaking clinical trials have demonstrated that AAV-hOTOF can restore hearing in child patients with severe DFNB9 deafness to near-normal levels, with significant improvements in auditory thresholds, pure-tone audiometry, and speech recognition abilities (Lv et al., 2024; Qi et al., 2024). However, patient age is a critical determinant in gene therapy outcomes. Preclinical and clinical studies predominantly select infant and young child subjects due to their greater neuroplasticity and attenuated immune responses, which correlate with superior hearing restoration potential. While this presents a significant barrier for treating ARHL, emerging clinical evidence suggests this challenge may be addressable. A recent multicenter clinical trial demonstrated effective hearing recovery in a 23.9-years-old DFNB9 deafness patient following AAV-OTOF injection (Qi J. et al., 2025). This finding indicates that adult patients can also derive significant benefit from gene therapy. Compared to cochlear implantation, gene therapy may offer more natural hearing restoration, potentially paving the way for novel therapeutic approaches to ARHL. Meanwhile, novel therapeutic strategies targeting hair cell regeneration–such as modulation of the Notch and Wnt signaling pathways–have shown promise in preclinical studies (Samarajeewa et al., 2019). While gene and cell-based therapies offer substantial potential for restoring natural hearing and targeting the underlying mechanisms of ARHL, their clinical translation remains highly challenging. In particular, gene therapy and stem cell therapy, though promising, are still in the early stages of development and face significant hurdles. The application of gene therapy to the human inner ear is constrained by several factors, including inefficient gene delivery, potential off-target effects, vector-induced immune responses, long-term stability of gene expression, and unresolved ethical issues. Similarly, stem cell-based approaches must overcome obstacles related to cell survival, integration, and functional maturation within the complex cochlear environment. These scientific, technical, and ethical limitations currently hinder widespread clinical adoption. Therefore, sustained research efforts are critical to improving delivery strategies, ensuring safety and specificity, and addressing ethical considerations–ultimately paving the way for the successful clinical implementation of these advanced therapeutic modalities.

### 5.4 Physical exercise

Physical exercise has been shown to provide robust and reproducible geroprotective effects in model organisms as well as in humans, and is widely recognized as one of the most effective interventions for enhancing healthspan and longevity (Furrer and Handschin, 2023). While the systemic benefits of exercise–such as improved cardiovascular, metabolic, and cognitive function–are well established, recent research has begun to extend these observations to auditory aging, suggesting that exercise may also play a role in preserving auditory function in later life. Epidemiological studies have reported that lower levels of physical activity are associated with a higher risk of hearing loss among older adults (Kuo et al., 2021; Martinez-Amezcua et al., 2021). In contrast, a valuable animal study has demonstrated that long-term, regular physical exercise can significantly delay the progression of ARHL (Han et al., 2016). Specifically, long-term voluntary wheel running was found to mitigate the age-related increase in ABR thresholds, preserve cochlear hair cells, SGNs and strial capillaries, and reduce cochlear inflammation–a key contributor to auditory degeneration during aging (Han et al., 2016). These findings suggest that exercise may exert its protective effects through anti-inflammatory, neuroprotective, and vascular-supporting mechanisms within the cochlea. However, further studies are needed to confirm these effects in human populations, particularly to determine the optimal type, intensity, and duration of exercise required to achieve auditory benefits. Additionally, research should aim to elucidate the molecular pathways by which physical activity influences cochlear aging, which may ultimately inform the development of combined lifestyle and pharmacological interventions for ARHL.

## 6 Translational gap between animal models and human applications

Despite the invaluable insights provided by animal models in auditory research, significant translational challenges remain in applying these findings to human ARHL. Fundamental interspecies differences in genetic background, anatomical structure, physiological function, lifespan, and disease progression often result in therapies that perform well in animals but prove less effective–or even ineffective in human clinical settings. These challenges are further compounded by the artificial nature of experimental conditions, limitations in study scale and duration, and the inherent biological variability between species. A striking example of this translational gap is highlighted by a study involving 120 human inner ear autopsy samples, which revealed that ARHL in humans is primarily driven by irreversible hair cell damage and loss. In contrast, degeneration of the stria vascularis–a central pathological focus in many animal models–did not show a significant correlation with hearing thresholds in humans (Wu et al., 2020). Moreover, the extent of cochlear sensory cell loss in human subjects, particularly in low-frequency regions, far exceeds that observed in any established aging animal model, underscoring a fundamental divergence in disease pathology. Neurodegeneration also demonstrates species-specific characteristics. For example, studies in aged rhesus monkeys have shown that hearing loss is predominantly neural in origin, with significant degeneration of SGNs, despite the preserved function of OHCs (Sun et al., 2021). In contrast, the widely used C57BL/6 mouse model exhibits a mixed phenotype, showing both hair cell loss and SGN degeneration (Jeng et al., 2020). These differences suggest that individual animal models may only reflect specific subtypes of ARHL–sensory, neural, or mixed–thus failing to capture the full clinical heterogeneity observed in humans. Additionally, the rate and pattern of cochlear aging vary markedly across species. Rodents, for example, undergo accelerated auditory degeneration due to their shorter lifespans, which may not accurately reflect the gradual, multifactorial decline seen in human aging. This temporal mismatch complicates the assessment of long-term therapeutic efficacy and safety in preclinical studies. Collectively, these findings emphasize a critical translational gap: no single animal model can fully recapitulate the complex and heterogeneous pathophysiology of human ARHL. As a consequence, promising therapeutic interventions identified in animal studies frequently fail during clinical trials due to unaccounted biological and mechanistic discrepancies.

Bridging this gap will require the development and integration of more human-relevant experimental platforms. These include the use of non-human primates, human inner ear organoids, and patient-derived induced pluripotent stem cell (iPSC) models, which offer greater fidelity to human biology (Koehler et al., 2017; Sun et al., 2025). Furthermore, emerging technologies such as organ-on-chip systems (Hu et al., 2024), computational modeling, and artificial intelligence hold significant potential to complement traditional models by enabling more predictive, scalable, and mechanistic assessments. While animal models are still essential for understanding disease mechanisms and developing treatments, their limitations need to be carefully considered. To improve the translational success of ARHL research, it is important to use a variety of models and to validate findings using human tissues and clinical data.

## 7 Conclusion

In conclusion, while significant progress has been made in understanding the mechanisms of ARHL, numerous challenges remain, particularly in the development of targeted treatments and interventions. (1) ARHL is influenced by a complex interplay of factors, how these mechanisms interact to cause hearing loss deserves to be explored. (2) Physiological differences of rodents from humans present substantial limitations, developing more accurate animal models that better mimic human physiology will enhance the translational potential of mechanistic research. (3) Pharmacological interventions, including anti-inflammatory and antioxidants, have shown promise, but their clinical efficacy and safety still require further validation. (4) Gene therapy and cell therapy, offer exciting possibilities to repair genetic mutations or damaged cochlear cells, potentially reversing or slowing ARHL. However, ethical, technological, and safety concerns need to be addressed before these therapies can be widely applied. (5) Non-pharmacological interventions, particularly regular physical exercise, have emerged as a valuable strategy to mitigate the progression of ARHL. But more comprehensive clinical studies are needed to determine the optimal types and intensities of exercise for individuals with ARHL. Future research should emphasize interdisciplinary collaboration such as genetics, molecular biology, pharmacology, and bioengineering to further uncover the complex mechanisms of ARHL. Integrating genetic, environmental, and clinical data will be crucial for developing personalized prevention and treatment strategies. A holistic approach that combines genetic insights, lifestyle modifications, and innovative therapeutic interventions will be key to advancing the management and treatment of ARHL.
